# Role of local complement activation in kidney fibrosis and repair

**DOI:** 10.1172/JCI188345

**Published:** 2025-06-16

**Authors:** Didier Portilla, Vikram Sabapathy, Daniel Chauss

**Affiliations:** 1 Department of Medicine and Center for Immunity and Regenerative Medicine, University of Virginia, Charlottesville, Virginia, USA.; 2 Immunoregulation Section, Kidney Diseases Branch, National Institute of Diabetes and Digestive and Kidney Diseases, NIH, Bethesda, Maryland, USA.

## Abstract

The complement system is an important component of the innate immune system involved in host defense and maintaining homeostasis. While the liver is the main source of complement proteins in the bloodstream, recent research has shown that various tissues, including the kidneys, can produce complement components locally in response to both acute and chronic inflammation. This Review highlights evidence from animal models of glomerular and tubulointerstitial kidney disease showing increased expression of intracellular complement in the kidneys. Studies using knockout mice for complement and complement receptors, along with complement inhibitors, have demonstrated that reduced complement activation in animal models of kidney fibrosis led to reduced inflammation and fibrosis, thereby supporting the pathogenic role of complement activation. Data from single-cell RNA-sequencing, spatial transcriptomics, and proteomics studies further demonstrate that alterations in local complement levels contribute to the fibrotic microenvironment observed in these models. Additionally, kidney biopsy results from patients with acute kidney injury and chronic kidney disease (CKD) indicate an increased expression of intracellular complement components as disease progresses. Developing drugs aimed at diminishing the expression and activation of local complement in glomerular and tubulointerstitial kidney disease could provide a novel approach to managing CKD.

## Introduction

**Figure 1:**
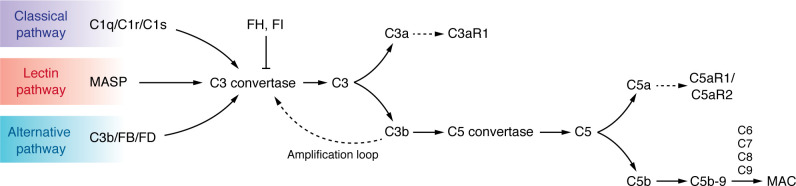
Overview of complement activation. The three complement activation pathways — classical, lectin, and alternative — converge at C3 convertase formation. C3 convertase cleaves C3 into C3a (inflammatory mediator) and C3b (opsonin), which participates in C5 convertase formation. C5 convertase cleaves C5 into C5a (inflammatory mediator) and C5b, which initiates the membrane attack complex (MAC) by recruiting C6, C7, C8, and C9. Regulatory proteins like factor H (FH) and factor I (FI) control excessive complement activation. The complement system enhances immune defense through inflammation, opsonization, and direct cell lysis via MAC formation. MASP, mannose-binding lectin-associated serine protease.

The complement system is a key component of the innate immune system activated in response to sterile and nonsterile inflammation (1). In humans, this system includes more than 40 soluble proteins and membrane-bound receptors in the bloodstream (2, 3). Although most complement proteins are produced by the liver (4), complement proteins can be synthesized locally in kidney tissue in response to injury and inflammation. The complement system consists of a series of proteolytic reactions that when activated operate through three pathways: the classical, alternative, and lectin pathways (Figure 1). This activation leads to the formation of multiprotein complexes known as C3 and C5 convertases. The subsequent degradation of these complexes produces anaphylatoxins C3a and C5a that play a role in the synthesis of the membrane attack complex (MAC), ultimately resulting in cell lysis (2, 5). Anaphylatoxins C3a and C5a are regulators of local and systemic inflammation through binding cognate receptors C3aR, C5aR1, and C5aR2 (6). While dysregulation of the complement system is well established in rare kidney diseases such as C3 glomerulopathy and atypical hemolytic uremic syndrome, recent evidence suggests that uncontrolled complement activation may also contribute to the progression of tubulointerstitial kidney disease in the transition from acute kidney injury (AKI) to chronic kidney disease (CKD), as well as in glomerular diseases, such as focal segmental glomerulosclerosis (FSGS) and diabetic kidney disease (DKD) (7–9).

CKD is a progressive condition characterized by a reduction in kidney function lasting for over 3 months. It affects more than 10% of the global population and contributes to significant morbidity and mortality (10, 11). A major risk factor for CKD is AKI, which predisposes to long-term kidney damage. Survivors of AKI are at higher risk of developing CKD, and AKI also contributes to renal fibrosis and nonrenal organ damage. Regardless of the etiology, CKD results in tubulointerstitial fibrosis and glomerulosclerosis, both considered hallmark features of CKD progression. Fibrosis, defined as the abnormal accumulation of extracellular matrix (ECM) components, disrupts normal kidney architecture by affecting the tubulointerstitial compartment, glomeruli (leading to glomerulosclerosis), and the vasculature (arteriosclerosis), driving progressive decline in renal function. This fibrotic remodeling resembles normal wound healing, involving an inflammatory response with the release of pro-inflammatory mediators and the infiltration of immune cells into the damaged tissue. These signals activate resident fibroblasts, perivascular cells, and mesenchymal cells, which then differentiate into myofibroblasts, the primary producers of ECM (12). However, pathological fibrosis lacks targeted elimination of myofibroblasts, leading to their persistent proliferation and accumulation (13, 14). To date, no effective antifibrotic therapy exists to halt CKD progression.

Although local complement activation is frequently observed in kidney disease, it remains unclear whether this contributes to pathogenesis or merely reflects a secondary phenomenon. Notably, proteinuria itself can activate complement pathways. This Review will focus on the role of local complement activation in models of glomerulosclerosis that occur independently of immune complex deposition, such as FSGS and DKD. Furthermore, we will explore new findings implicating increased complement C3 in failed-repair proximal tubule cells (PTCs) during the AKI-to-CKD transition as a driver of tubulointerstitial fibrosis. Emerging data from the Kidney Precision Medicine Project (KPMP) highlight that several complement components, including C3 in maladaptive PTCs, C3aR1 in macrophages, and complement factor H (CFH) in myofibroblasts, are upregulated in kidney tissue from patients with AKI and CKD. While further research is required to clarify the causative role of complement activation in fibrosis, studies in mouse models support the therapeutic potential of targeting local complement pathways to ameliorate tubulointerstitial disease and progressive CKD.

## Complement activation in glomerulosclerosis

In this section, we discuss experimental findings from animal models that suggest that increased proteinuria triggers the de novo synthesis of markers of complement activation, including complement C3 and soluble C5b-9 (sC5b-9) in the proximal tubule. Clinical studies and murine models have shown that increased proteinuria accelerates the progression of kidney disease. This indicates that proteinuria disrupts the normal balance between reabsorptive and secretory functions of the kidney. Additionally, proteinuria leads to increased production of PTC cytokines and complement deposition of C3 and C5b-9, resulting in inflammatory cell infiltration and increased fibrogenesis in the tubulointerstitial compartment (15).

One possible explanation for these changes is that the disruption of the glomerular filtration barrier in proteinuric kidney diseases allows proteins, including complement factors, to leak into the urine. Nonselective proteinuria, characterized by the loss of high–molecular weight proteins, is associated with elevated urinary levels of sC5b-9, a marker of complement activation. This activation contributes to increased tubulointerstitial injury and fibrosis (16). Studies in proteinuric animal models have shown that complement depletion reduces tubulointerstitial injury, supporting the role for complement in tubulointerstitial disease due to proteinuria (17, 18).

Recent research in kidney transplant patients with nonselective proteinuria has demonstrated the deposition of C3dg and C5b-9 at the apical side of PTCs (19). These findings were supported by animal and in vitro studies of PTCs exposed to nonlytic doses of serum proteins (20). The susceptibility of the apical side of the PTCs to complement activation has been attributed to low expression of complement regulatory proteins on this surface (21).

A recent study demonstrated that the urokinase-type plasminogen activator (uPA) activates complement with formation of C3a and C5a anaphylatoxins in urine from healthy persons when exogenous, inactive, plasminogen, and complement factors are added. This effect was inhibited by the potassium-sparing diuretic amiloride. In the study these findings in human urine were corroborated in podocin-deficient mice, an animal model of glomerular proteinuria. The investigators found that in these mice, amiloride and the use of uPA antibodies also decreased urine anaphylatoxin excretion with reduced inflammation but not reduced fibrosis. These results indicate that active plasmin contributes to the generation of anaphylatoxins in tubular fluid of mice with glomerular proteinuria but also suggest that additional mechanisms other than complement anaphylatoxins could contribute to the development of fibrosis in this animal model (22).

In the following section, we will briefly discuss experimental evidence illustrating the role of complement activation in glomerular injury not associated with immune complex deposition, such as FSGS and DKD. Recent studies in these two glomerular diseases have also reported increased PTC C3 expression associated with progressive kidney disease. Detailed reviews on the involvement of complement activation in immune complex glomerulopathies, such as IgA nephropathy, C3 glomerulonephritis, and lupus nephritis (LN), have been recently published (8, 23–25).

### Local complement activation in DKD.

DKD is the leading cause of CKD and end-stage renal disease (26). It is triggered by prolonged exposure to elevated glucose levels, resulting in podocyte dysfunction, proteinuria, and glomerulosclerosis. Previous studies (27) have shown increased urinary proteins enriched in the alternative complement pathway in patients with DKD and proteinuria. Additionally, deposition of C1q and C3c in glomeruli has been reported in patients with DKD, correlating with proteinuria and glomerular lesions (28).

A genome-wide transcriptome analysis of a diverse group of patients with DKD revealed differential regulation of the canonical complement signaling pathway, including complement C3, in both DKD glomeruli and tubulointerstitial compartments (29). Two main mechanisms explain the involvement of complement in DKD: triggering of the lectin pathway by glycated proteins and impaired function of complement regulatory proteins, such as CD59, due to glycation (30–32).

While immune-mediated inflammation including complement activation plays a role in podocyte injury in DKD, the precise cellular mechanisms involved are not entirely clear. Recent research supports the involvement of alternative complement activation in podocyte dysfunction in patients with DKD. This study observed increased protein expression of complement components complement factor B (CFB), C3d, C5b-9, and C5aR in DKD glomeruli and tubules (33) both in patients with DKD and in diabetic mice. To define the role of CFB in podocyte injury, the investigators used CFB shRNA and CFB-deficient mice. They demonstrated that CFB knockdown inhibits the renal alternative complement pathway, resulting in reduced protein expression of CFB, with significant reduction in podocyte injury, as well as in clinical and histological parameters of DKD. Their findings support the presence of local CFB synthesis contributing to podocyte injury yet cannot rule out that CFB shRNA could have affected systemic CFB production by the diabetic state in mice. An important contribution of this study is that the investigators also defined potential upstream pathways by which CFB could be stimulated by high glucose in DKD. They found that mTOR complex 1 (mTORC1) signaling was activated, and blockade of mTORC1 signaling with rapamycin abolished alternative complement pathway activation in podocytes. In addition, activation of podocyte mTORC1 signaling led to STAT1 phosphorylation, which in turn upregulated CFB expression. The study suggests that CFB could be a therapeutic target because CFB deficiency reduced inflammation — measured as reduced NF-κB activation — and reduced ceramide synthesis, also leading to reduced tubular cell damage. Another study performed single-nucleus RNA-Seq (snRNA-Seq) and single-nucleus assay for transposase-accessible chromatin with sequencing (snATAC-Seq) on kidney cortex samples from patients with type 2 diabetes to identify cell-specific differentially expressed genes and accessible chromatin regions associated with DKD. The findings revealed an increased number of VCAM-, hepatitis A virus cellular receptor 1–, and complement C3–positive proximal tubules and infiltrating immune cells in DKD samples compared with controls (34).

Although these studies shed light on complement activation in DKD, it remains unclear whether complement activation is primarily driven by the diabetic environment or influenced by other factors. Both studies suggest potential therapeutic targets to mitigate complement activation and progressive DKD.

### Complement activation in FSGS.

FSGS is a complex disease associated with podocyte injury leading to increased glomerular proteinuria with progressive deterioration of kidney function (35). While FSGS is not typically associated with immune complex deposition, experimental evidence has shown that some patients with FSGS exhibit glomerular deposition of IgM, triggering classical complement activation (36).

Studies using the adriamycin nephropathy model of FSGS have demonstrated that kidney damage in FSGS is dependent on the local production of C3 (37). PTCs are found to produce C3 and secrete inflammatory cytokines, such as TNF-α and IL-6, and monocyte chemoattractant protein–1, which recruit and activate macrophages in the tubulointerstitial compartment. These macrophages release pro-inflammatory and pro-fibrotic cytokines, ECM-remodeling factors, and collagens, contributing to further kidney damage. Additionally, in vitro studies have shown that stimulation of human PTCs in culture with normal human serum can also spontaneously activate complement by the alternative pathway, leading to binding of C5b-9 to the cell surface. These events are followed by marked cytoskeleton alterations (38).

Studies by Turnberg et al. (39) have revealed that mice deficient in complement C3 or factor D and treated with adriamycin show reduced fibrosis when compared with wild-type mice, highlighting the importance of the alternative pathway activation in this model.

However, conflicting results were obtained with the use of CFB-deficient mice, where CFB deficiency failed to protect against fibrosis (40).

Another study using the adriamycin model of FSGS found that loss of decay-accelerating factor (CD55) on podocytes initiates complement C3aR signaling, leading to IL-1β production and subsequent podocyte injury, implicating C3a/C3aR signaling in the pathogenesis of FSGS (41). This finding supports the potential use of C3a/C3aR or IL-1β/IL-1R1 inhibition to reduce progression of proteinuric nephropathies.

Further research by Stepanova et al. (42) has investigated the role of renal epithelial complement C3 expression in the progression of kidney fibrosis in FSGS. Although the stage of CKD was not clearly defined in this study, increased C3 mRNA and positive immunostaining for C3 in tubules were observed in patients with FSGS compared with healthy controls, supporting the role of complement activation in the progression of this kidney disease.

## Complement activation in tubulointerstitial fibrosis

Current evidence supports the concept that primary glomerular injury can lead to tubulointerstitial injury and progressive decline in kidney function. However, injury to proximal tubules during AKI can also contribute to damage in podocytes and parietal epithelial cells, ultimately promoting the progression of kidney disease (43). AKI is now recognized as a significant risk factor for the development of CKD (44). This insight has shifted research focus toward understanding the mechanisms of progressive scarring following tubular injury. In the context of AKI, tubulointerstitial disease is associated with increased complement activation. Although the exact cellular mechanisms remain incompletely understood, elevated expression of C1q in the kidney interstitium and complement component C3 in the proximal tubule appear to contribute to this pathological process.

C1q, a protein involved in initiating the classical complement pathway, was recently shown to participate in various cellular processes independent of complement activation (45). As a pattern recognition molecule of the classical pathway, C1q binds a broad range of self- and non-self-ligands via its heterotrimeric globular C1q (gC1q) domain (46). Notably, the trimeric gC1q signature domain has been identified in a variety of noncomplement proteins, collectively classified as the C1q family (47). Interestingly, crystal structures of the gC1q domain from C1q family members reveal a compact architecture resembling that of the multifunctional TNF ligand family (48). Together, these proteins form the C1q and TNF superfamily, which has been implicated in the pathogenesis of kidney injury.

In key experiments using mouse models of CKD, including 5/6 nephrectomy, folic acid (FA) nephropathy, and unilateral ureteral obstruction (UUO) models, investigators observed increased complement activation in kidney tissue as well as in perivascular and immune cells. This was accompanied by upregulated expression of C1q, C3 fragments, C5, and complement receptors C3aR and C5aR, along with the presence of tubulointerstitial fibrosis. Specifically, C1q was found to be upregulated in interstitial cells, including macrophages and stromal cells. Notably, reduced C3 expression achieved through the use of C3-deficient mice was associated with decreased inflammation and reduced kidney fibrosis across all injury models, suggesting a direct role for C3 and C1q in the development of kidney fibrosis (49, 50).

Furthermore, FA-treated mice with global deletion of C1r exhibited reduced inflammation and kidney fibrosis when compared with FA-treated control mice (51). In human studies, biopsy samples from patients with acute tubular necrosis and acute interstitial nephritis revealed a distinct set of inflammation-associated genes encoding C1Q subunits (C1QA and C1QB) and CD163, a marker of kidney-resident macrophages (52). The authors concluded that the expansion of reprogrammed C1Q^+^CD163^+^ macrophages, regardless of AKI etiology, is a conserved feature across both mouse and human species and contributes to kidney injury.

We recently presented spatial transcriptomics analysis of kidney tissue from mouse models of ischemia/reperfusion injury (IRI) and UUO that further support these findings. Specifically, we observed increased expression of C1q in kidney interstitial cells, predominantly within macrophages and stromal cells (53).

### Increased PTC C3 expression in AKI-to-CKD transition.

Previous clinical studies have established a strong association between AKI and the subsequent development of CKD (54–56). This link has been further validated by research in mouse models (57). However, the pathophysiological mechanisms driving the AKI-to-CKD transition remain poorly understood, largely due to the complexity and heterogeneity of the kidney’s cellular composition involved in AKI-to-CKD transition and the limitations of existing mouse models of CKD (58). Despite these challenges, PTCs have emerged as key players in this transition, prompting focused efforts to elucidate the mechanisms underlying their contribution to disease progression. PTCs express important solute transporters and are particularly vulnerable to injury because of their high metabolic demands. During AKI, whether triggered by ischemia or toxins, PTCs may suffer sublethal damage or undergo cell death due to intracellular ATP depletion. Several recent studies have advanced our understanding of how PTCs fail to repair following ischemic injury.

**Figure 2:**
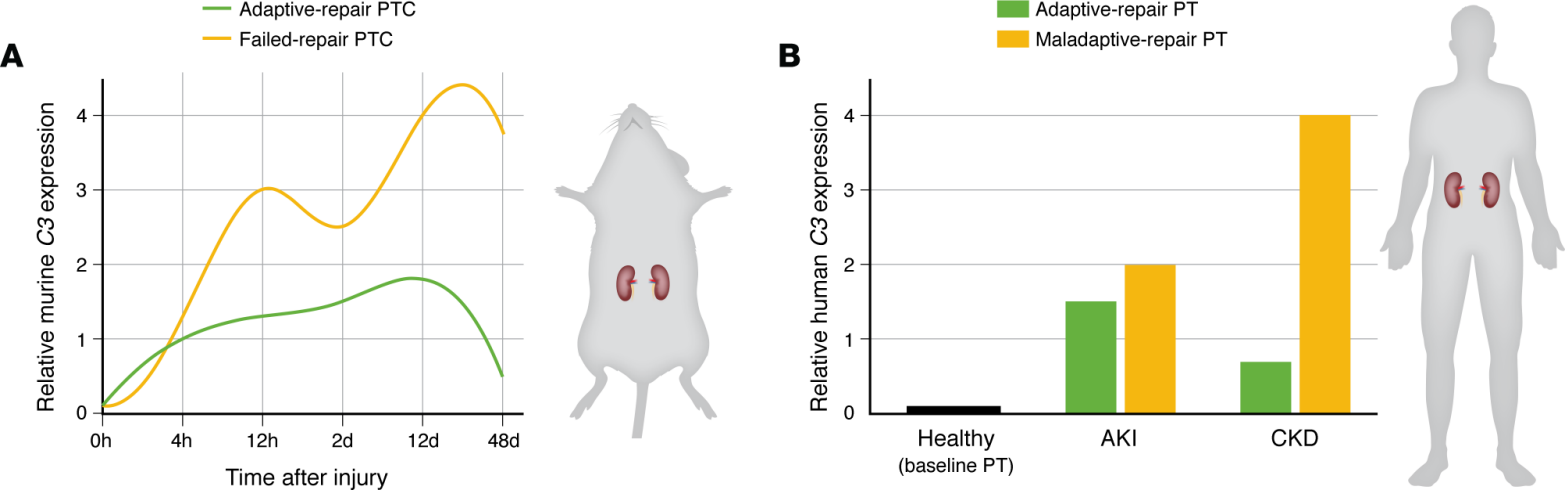
*C3* expression dynamics correspond to failed repair in proximal tubules. (**A**) Temporal kinetics of murine *C3* expression during tubular repair following AKI (bilateral ischemia). Smoothed expression trajectories of *C3* in proximal tubular cells (PTCs) during kidney injury and repair. Murine data (from datasets published in ref. 59) indicate a biphasic induction of *C3* expression, with markedly increased and sustained expression in failed-repair PTCs compared with adaptive-repair PTCs. To represent the patterns of expression, dynamics were modeled using spline interpolation across six time points (0 hour, 4 hours, 12 hours, 2 days, 12 days, and 48 days) following injury. (**B**) Enhanced *C3* gene activity in maladaptive proximal tubular states in human kidney disease. Relative *C3* gene activity across proximal tubular subsets derived from human kidney single-cell sequencing data in healthy controls, AKI, and CKD (see ref. 65; datasets available from the KPMP, https://www.kpmp.org, and analyzed here with permission). Proximal tubular cells (PTs) from healthy kidneys exhibit minimal *C3* activity, whereas *C3* expression is substantially elevated in maladaptive repair PTs across both AKI and CKD settings. Bar height reflects relative gene activity.

Kirita et al. (59), using a bilateral ischemia model and snRNA-Seq, analyzed mouse kidney tissue at multiple time points (4 and 12 hours and 2, 4, 14, and 42 days after AKI). They identified a distinct population of PTCs that failed to undergo proper repair. While successfully repairing PTCs demonstrate early upregulation of growth factors, cytokines, and cell cycle genes, failed-repair PTCs express a unique set of secreted pro-inflammatory vasoconstrictive molecules, including endothelin and TGF-β. Notably, these cells also show increased expression of inflammation-related genes, such as *Vcam1*, *Ccl2*, and *C3*, at 14 days, a pattern persisting through day 42 (summarized in Figure 2A). These failed-repair PTCs exhibited reduced expression of terminal differentiation markers, including key transporters, and constituted at least 20% of injured tubules. Similar observations were reported in an FA-induced nephropathy mouse model (60).

In a follow-up study, Muto et al. (61) used snATAC-Seq to investigate cell-specific epigenetic landscapes and gene regulatory changes during the AKI-to-CKD transition in mice. This approach verified the cellular identity and chromatin accessibility of genes identified in prior snRNA-Seq studies. With a focus on *C3* and *Vcam1* expression, chromatin accessibility profiles were analyzed across several time points (sham, 4 and 12 hours, at day 2, 14, and day 42) and across annotated PTC subpopulations, including healthy, acutely stressed, injured, severely injured, and failed-repair states. Notably, gene activity for *C3* and *Vcam1* was significantly elevated in failed-repair PTCs at day 14, suggesting these genes play key roles in maladaptive repair and progression to CKD.

Complementary findings by Gerhardt (62, 63) using the ischemic AKI model further characterized distinct injured PTC states. These studies implicated NF-κB and activator protein 1 pathway activity in driving a transition of PTCs to a pathological phenotype. Similar to the Kirita study, a unique cluster of PTCs was identified, marked by high expression of both VCAM1 and complement C3, representing a pro-inflammatory, pro-fibrotic population with features of cellular senescence.

Emerging evidence suggests that damaged PTCs play an active role in modulating the local microenvironment through interactions with immune and stromal cells. Recent spatial transcriptomics analyses have identified high intracellular levels of complement C3 in leukocytes located in close proximity to failed-repair PTCs (64). Our own spatial transcriptomics data from mouse models of IRI and UUO support these findings, revealing persistently injured, dedifferentiated PTCs with elevated mRNA and protein expression of complement C3 and CFB. These findings suggest that local complement activation, particularly via the alternative pathway, is central to sustaining inflammation and fibrosis in the injured kidney. The spatial association of failed-repair PTCs with immune cells and stromal components highlights a pathophysiological niche in which complement-mediated crosstalk likely drives the progression from AKI to CKD.

### Local complement activation in human kidneys.

Recent studies using kidney biopsies from patients with AKI and CKD have provided valuable insights into potential biomarkers and mechanisms by which kidney cells participate in the process of renal scarring. The KPMP (65) provides understanding of kidney diseases by creating an extensive repository of biopsies from adults with various types and severities of AKI and CKD (66). KPMP integrates kidney tissue data from patients with AKI, patients with CKD, and healthy controls to develop a detailed kidney tissue atlas. This atlas is designed to help define disease subgroups and identify cell types, pathways, and therapeutic targets. Publicly available KPMP data sets include kidney biopsies from healthy controls (*n* = 28), patients with AKI (*n* = 14), and patients with CKD (*n* = 37).

To highlight complement C3 expression in injured proximal tubular epithelial cells across different kidney disease states, we analyzed the KPMP datasets (65). We identified cells annotated as adaptive/maladaptive/reparative PTCs (aPTs) based on clustering in the original KPMP metadata. As shown in Figure 2B, our examination of the datasets demonstrated that C3 expression was minimal in healthy controls but upregulated in aPTs during both AKI and CKD. The pattern suggests that C3 is activated in response to injury and remains elevated during maladaptive repair, implicating it in the progression of kidney disease. In addition to upregulated C3 expression in maladaptive proximal tubules, the KPMP data also show upregulation of complement receptor C3aR1 in the monocyte/macrophage population and upregulation of CFH in myofibroblasts in patients with AKI and CKD. While these findings do not establish a causal role for these complement components in kidney disease progression, these observations represent support in the existing data for the presence of a human failed-repair PTC population expressing high levels of C3, as well as increased inflammatory and pro-fibrotic complement components, such as C3aR1 and CFH, which may contribute to the pro-fibrotic renal microenvironment observed in AKI-to-CKD transition in mouse models.

In a complementary study, the KPMP consortium used integrated multiplexed proteomics, single-cell analysis, and spatial transcriptomics to further characterize failed-repair PTC populations in human kidney biopsies. This work revealed that kidney disease is associated with decreased expression of THY1, a mesenchymal stromal and stem cell marker that regulates PTC differentiation. Conversely, expression of prominin 1 (PROM1), another stem cell marker linked to failed-repair PTCs after AKI, was increased. PROM1 was localized near inflammatory niches while THY1 was reduced in CKD (67). The findings provide translational evidence consistent with animal model studies, reinforcing the presence of a dedifferentiated, nonreparative PTC population characterized by elevated C3 expression and its potential role in driving kidney fibrosis and disease progression.

### Complement receptor expression in kidney fibrosis.

The cleavage of complement components C3 and C5 by their respective convertases generates the biologically active anaphylatoxins C3a and C5a. Several studies suggest that C3a has both pro-inflammatory and antiinflammatory effects, depending on the cell types involved and disease context (68). C3a promotes cell migration, cytokine production, and histamine release (69), while inhibiting LPS release in nonadherent monocytes (70) and inducing phagocytosis by macrophages (71). C5a is a potent chemoattractant for neutrophils and macrophages and stimulates histamine release from mast cells, contributing to inflammation (72). C3a and C5a exert their functions by binding to their G protein–coupled receptors C3aR, C5aR1, and C5aR2, which are expressed on tubular epithelial cells and myeloid cells, including neutrophils, macrophages, and activated T cells (73, 74).

The roles of C3aR and C5aR1 signaling in kidney have been extensively studied in various animal models. In the adriamycin-induced proteinuric model where both glomerular and tubulointerstitial fibrosis are present, C3aR-deficient mice were protected from tubular injury, fibrosis, and renal failure compared with wild-type controls. These findings support a pathogenic role for C3aR in kidney damage. C3a has been shown to drive epithelial-mesenchymal transition in proteinuric nephropathy (75). However, these studies fall short of conclusively proving that local complement production by kidney-resident cells perpetuates inflammation because of the lack of experimental models using tissue-specific complement inhibition.

Further evidence comes from IRI models, where tubular epithelial cells were found to produce the chemokines MIP-2 (CXCL2) and KC (CXCL1) postinjury. In vitro, PTCs exposed to mouse serum produced both MIP-2 and KC, and selective C3aR antagonism significantly reduced production of these chemokines. In contrast, C5aR1 antagonism had minimal effect. This suggests that C3aR activation in PTCs mediates the synthesis of pro-inflammatory chemokines, potentially signaling systemic complement activation (76). Additional studies using C3aR- or C5aR1-deficient mice demonstrated protection against IRI-induced kidney injury, characterized by reduced immune cell infiltration and lower expression of kidney injury molecule KIM-1 and adhesion molecules (77), reinforcing the role of anaphylatoxin receptor activation in kidney damage.

Aristolochic acid nephropathy (AAN), a progressive interstitial fibrosis disorder initially reported in 1964 in Asia, has been linked to chronic ingestion of aristolochic acid–containing herbal remedies (78). In mouse models of AAN, increased expression of C3a and C3aR in tubular cells and macrophages was identified as a key contributor to tubulointerstitial fibrosis (79, 80). Similarly, in the UUO model, elevated C3aR expression in tubular epithelial cells was shown to modulate NLRP3 inflammasome activity in tubular epithelial cells, leading to enhanced IL-1β production and inflammation (81). Although inhibitors like SB290157 have demonstrated protection against kidney inflammation and fibrosis by blocking C3aR function (82), their clinical application is limited by findings that SB290157 may act as an agonist for both C3aR and C5aR2 (83).

Although prior reviews have emphasized the pathogenic role of anaphylatoxin receptor upregulation in kidney fibrosis (84), recent human studies provided further mechanistic insights, particularly for C5aR1 in LN. RNA-Seq of kidney tissue revealed increased expression of C5aR1 and dynamin related protein 1 (Drp1) in podocytes. Drp1, a key regulator of mitochondrial fission, was implicated in podocyte injury. Pharmacological inhibition of C5aR1, using the nonpeptide inhibitor avacopan, reduced mitochondrial fission and podocyte damage, identifying dysfunction in mitochondrial dynamics and C5aR1 signaling as key drivers of LN pathology (85).

In other models, including FA-induced nephropathy (86) and the 5/6 nephrectomy model (87), increased C5aR1 expression was observed in infiltrating immune cells. Selective deletion of C5aR1 in macrophages or treatment with the C5aR1 inhibitor avacopan reduced inflammation and fibrosis, suggesting a pathogenic role for immune cell–associated C5aR1. In contrast, studies using the AAN and FA nephropathy models found that kidney tubule–specific *C5ar1*-deficient mice exhibited worsened injury (88), suggesting a renoprotective role for tubular C5aR1. This aligns with earlier findings on the hepatoprotective effects of C5aR1 in liver regeneration (89).

Collectively these findings underscore the complex and context-dependent roles of anaphylatoxin receptors in kidney disease, with different effects depending on receptor type, cellular localization, and disease model.

## The fibrotic microenvironment in CKD

In this section, we review experimental data indicating that additional complement components contribute to the heightened inflammatory and fibrotic responses during the AKI-to-CKD transition. For instance, elevated expression of the complement receptor C3aR1 has been observed in infiltrating macrophages, while CFH expression is increased in myofibroblasts. Although these findings do not establish a direct causal link between complement activation and fibrosis, they suggest that multiple complement components are involved in the inflammatory and scarring processes associated with kidney injury. Future studies will require improved tools — such as advanced fate mapping and cell type–specific deletion of complement components — to define their precise roles in kidney injury and fibrosis.

In CKD, the fibrotic microenvironment (FME), also known as the fibrogenic niche, refers to a specialized cellular and ECM landscape within the kidney characterized by excessive ECM deposition, leading to scarring and loss of function. This niche comprises kidney-resident cells, including tubular epithelial cells, peritubular capillary endothelial cells, pericytes, interstitial fibroblasts, and infiltrating immune cells, as well as extracellular vesicles, soluble mediators, and a distinct ECM network (90, 91). The FME promotes immune cell infiltration and sustained inflammation. For example, connective tissue growth factor, a heparin-binding ECM protein elevated during fibrosis, promotes macrophage accumulation by upregulating chemokines and cytokines in the kidney (92). Similarly, periostin, another ECM protein induced in injured kidneys, mediates inflammation and fibrosis via β3 integrin–focal adhesion signaling (93–95).

Recent proteomic analysis of decellularized kidney tissue scaffolds from healthy and fibrotic kidneys identified several upregulated ECM components, including matricellular proteins, proteoglycans, and matrix-modifying enzymes. Among them, vitronectin (VTN), a glycoprotein, was found to be significantly upregulated in mouse models of CKD, including UUO and IRI models. VTN was secreted primarily by activated macrophages. Its levels were also elevated in the urine of patients with CKD and inversely correlated with kidney function. Notably, global deletion of *Vtn* in mice conferred protection against kidney fibrosis (96). Furthermore, VTN has been associated with complement activation in glomeruli from patients with phospholipase A2 receptor–associated membranous nephropathy, reinforcing its role in the fibrotic process (97).

Macrophage infiltration into the injured kidney plays a central role in fibrosis (98–100). Kidney macrophages consist of both embryonically derived tissue-resident populations and inflammatory monocyte-derived macrophages. These cells can adopt diverse phenotypes depending on the microenvironment: M1 macrophages are pro-inflammatory, while M2 macrophages are traditionally considered antiinflammatory and reparative (101). However, recent evidence indicates that this binary classification oversimplifies macrophage heterogeneity and dynamic roles in kidney disease progression (102).

Targeting macrophages, a major cellular component of the FME, represents a promising therapeutic strategy. A recent IRI study in mice identified CD206^+^ M2-like macrophages as pro-fibrotic, expressing genes such as *Fn1*, *Spp1*, *Arg1*, *Tnf*, and *C3aR1*. These macrophages were shown to be major drivers of fibrosis. The study also introduced a novel therapeutic approach using a 12-mer peptide, termed bioactivated in vivo assembly peptides, which selectively disrupted macrophage membranes and mitochondrial function, inducing cell death. In the IRI model, this peptide reduced fibrosis and preserved kidney function by reshaping the immune microenvironment, offering a potential therapeutic avenue for CKD (103).

Fibroblasts, another critical component of the FME, also play a central role in kidney fibrosis. A recent multiomic study integrated Visium whole-transcriptome spatial analysis with single-cell and single-nucleus transcriptomic and epigenomic data (scRNA-Seq, snRNA-Seq, snATAC-Seq, and CosMx SMI) to characterize fibrotic stroma in human kidneys. This study defined not only fibroblast and myofibroblast populations but also endothelial cells contributing to FME organization, which resembled lymphoid structures near injured peritubular capillaries. Importantly, gene signatures associated with specific microenvironments — including the FME — were identified and may serve as predictive markers of kidney function decline in patients with CKD (104). Interestingly, the FME gene signature included complement-related genes, including CFH, which was also identified as a marker of proliferating myofibroblasts and parietal epithelial cells.

Supporting this, previous studies in the UUO and cisplatin-induced fibrosis models have reported increased CFH expression in myofibroblasts (105, 106). While CFH is classically known as a negative regulator of the alternative complement pathway, recent findings suggest it may also have noncanonical roles. One study revealed an intracellular function of CFH in tumor cells, including clear cell renal cell carcinoma and lung adenocarcinoma, where it regulated transcriptional activity and promoted proliferation, motility, and migration, functions not observed in normal or squamous carcinoma cells (107). These findings raise the possibility that elevated CFH expression in myofibroblasts during kidney fibrosis may similarly reflect a noncanonical, pro-proliferative role. A recent study (108) reported that CFH deficiency in lung fibrosis enhanced macrophage efferocytosis and autophagy and reduced macrophage-mediated inflammation and fibrosis. These findings suggest that CFH could be a potential therapeutic target in organ fibrosis.

**Figure 3:**
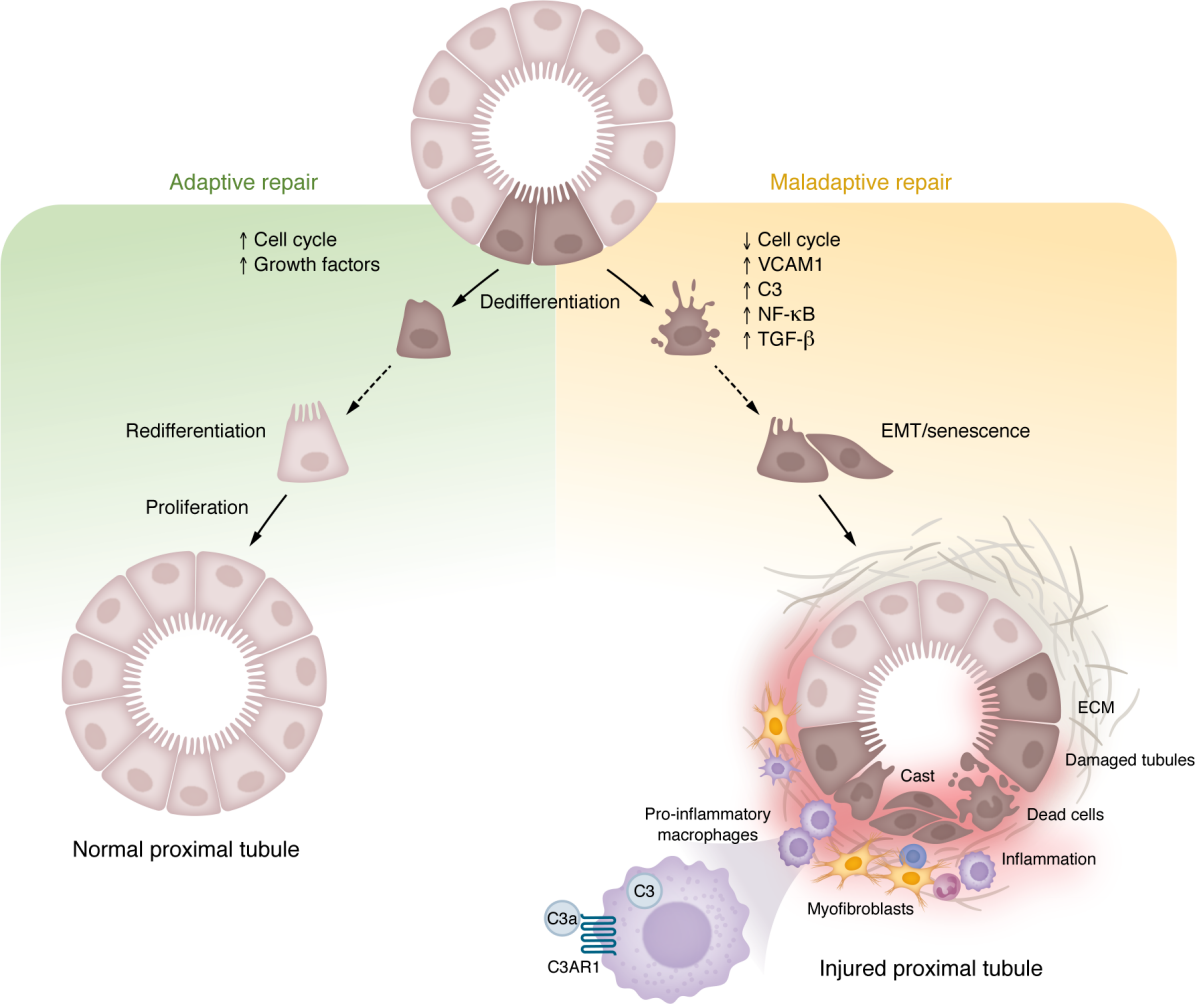
Cellular response of PTCs to injury. Ischemic or nephrotoxic insult activates pathways leading to either adaptive or maladaptive repair. Adaptive repair is characterized by dedifferentiation, proliferation, and redifferentiation, facilitating functional recovery. In contrast, maladaptive repair is marked by prolonged cell cycle arrest, and upregulation of VCAM1, C3, NF-κB, and TGF-β, among others, leading to epithelial-mesenchymal transition (EMT), senescence, fibrosis, and inflammation. The maladaptive response is further exacerbated by pro-inflammatory macrophage activation via C3AR1, contributing to sustained inflammation and ECM deposition. This model underscores the dichotomy between successful tubular regeneration and progressive kidney fibrosis, implicating C3-driven immune modulation in maladaptive repair.

Together, these data underscore the complexity of the FME and the interplay between tubular epithelial cells, immune cells, fibroblasts, and complement components in driving kidney fibrosis (Figure 3). Targeting components of the FME, including complement regulators like CFH, macrophage subtypes, or ECM modifiers holds promise for the development of novel antifibrotic therapies in CKD.

## Future perspectives on complement inhibition–directed therapies against CKD

A deeper understanding of the multifaceted roles that complement proteins play in the development of fibrotic kidney disease opens opportunities for targeted therapeutic strategies aimed at reducing CKD progression. While better addressing the underlying mechanisms driving CKD, the routine implementation of high-throughput assays, including single cell, and spatial transcriptomics will likely uncover complex associations involved in the regulation and downstream consequences of renal complement activation. By integrating multiomic strategies — such as epigenomic, proteomic, and 3D spatial analyses — we could further elucidate the intricate multicellular relationships that regulate renal complement activity. These high-dimensional data will be instrumental in discovering future CKD therapies, as demonstrated by recent clinical trials suggesting that novel C3 or CFB inhibitors can effectively target cells involved in renal fibrosis. However, further development of targeted delivery systems is necessary to introduce complement modulators specifically to the relevant cells or cell states in situ, thus mitigating those potentially detrimental side effects due to complement inhibition.

In conclusion, a variety of kidney diseases that lead to CKD are characterized by significant complement activation across diverse cell types. Both clinical studies and experimental evidence strongly suggest that locally produced complement proteins could be harnessed as therapeutic targets to ameliorate progression of kidney disease.

## Version 1 06/16/2025

Electronic publication
